# A transgenic approach for controlling *Lygus* in cotton

**DOI:** 10.1038/ncomms12213

**Published:** 2016-07-18

**Authors:** Anilkumar Gowda, Timothy J. Rydel, Andrew M. Wollacott, Robert S. Brown, Waseem Akbar, Thomas L. Clark, Stanislaw Flasinski, Jeffrey R. Nageotte, Andrew C. Read, Xiaohong Shi, Brent J. Werner, Michael J. Pleau, James A. Baum

**Affiliations:** 1 Monsanto Company, Chesterfield, Missouri 63017, USA

## Abstract

*Lygus* species of plant-feeding insects have emerged as economically important pests of cotton in the United States. These species are not controlled by commercial *Bacillus thuringiensis* (Bt) cotton varieties resulting in economic losses and increased application of insecticide. Previously, a Bt crystal protein (Cry51Aa2) was reported with insecticidal activity against *Lygus* spp. However, transgenic cotton plants expressing this protein did not exhibit effective protection from *Lygus* feeding damage. Here we employ various optimization strategies, informed in part by protein crystallography and modelling, to identify limited amino-acid substitutions in Cry51Aa2 that increase insecticidal activity towards *Lygus* spp. by >200-fold. Transgenic cotton expressing the variant protein, Cry51Aa2.834_16, reduce populations of *Lygus* spp. up to 30-fold in whole-plant caged field trials. One transgenic event, designated MON88702, has been selected for further development of cotton varieties that could potentially reduce or eliminate insecticide application for control of *Lygus* and the associated environmental impacts.

## Abstract (web summary)

Plant-feeding insects of the *Lygus* genus have emerged as a major pest effecting cotton crops in the USA. Here the authors optimize the insecticidal activity of a *Bacillus thuringiensis* crystal protein and produce transgenic plants that are resistant to feeding damage by *Lygus* species.

## Graphical abstract

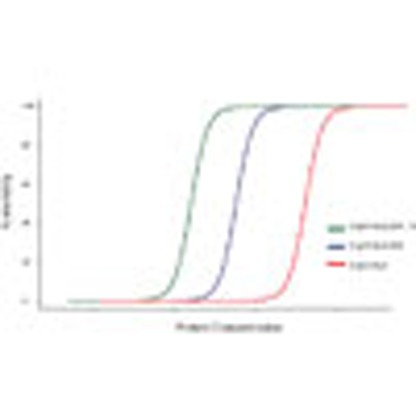

The development and cultivation of transgenic crops has revolutionized agriculture worldwide. In 2014, the global cultivation of insect-protected crops was estimated at 78.8 M ha (ref. 1). These crops express *Bacillus thuringiensis* (Bt) insecticidal proteins, providing excellent control of economically important coleopteran and lepidopteran pests, but are not effective in controlling hemipteran pests. Bt cotton provides effective control of lepidopteran pests resulting in the increased yield and reduced application of broad spectrum synthetic insecticides. Other benefits include area-wide suppression of lepidopteran pest species in other crops^2^ and a resurgence in beneficial insects for improved integrated pest management^3^. In this low spray environment conferred using Bt crops, some pests such as plant bugs have emerged as important economic pests of cotton in many countries^4^,^5^. In the United States, the plant bugs *Lygus hesperus* Knight and *L*. *lineolaris* (Palisot de Beauvois; Hemiptera: Miridae) have attained status as the two most economically damaging pests of cotton^6^. Nymph and adult *Lygus* spp. feed on numerous plant species including major row crops, inflicting damage by direct feeding^7^. On cotton, *Lygus* feeding on small- to medium-sized squares cause abscission resulting in yield loss^7^, while feeding on large squares results in damage to anthers leading to abnormal flowers.

We previously reported the isolation and characterization of a *Bt* crystal (Cry) protein, TIC807, with insecticidal activity towards both *L. hesperus* and *L*. *lineolaris*^8^. This 35-kDa protein belongs to the β pore-forming protein family of bacterial toxins and has been designated Cry51Aa2 (accession GU570697) according to the established *Bt* protein nomenclature^9^. Cotton plants expressing Cry51Aa2 reduced the survival and development of *L. hesperus* nymphs compared with the non-traited control, but exhibited less control of *L*. *lineolaris*; neither control was commercially acceptable^8^.

Successful development of insect-protected crops often requires modification of insecticidal proteins for improved activity. Such modifications can result in increased potency, improved spectrum or both, and have been achieved through various approaches such as the exchange of domains between various insecticidal proteins, addition of proteolytic active sites and targeted amino-acid substitutions. Examples of modified proteins expressed in commercially available insect-protected crops include eCry3.1Ab (ref. 10), mCry3Aa (ref. 11), Cry3Bb (ref. 12) and Cry1A.105 (http://Cera-gmc.org/index.php?action=gm_crop_database&mode=showProd&data=MON89034), all members of the three-domain family of crystal proteins, the best known group of crystal proteins found in Bt. In this study, we extend this approach to the β pore-forming protein, Cry51Aa2, for the purpose of controlling hemipteran pest species. We report modifications made to Cry51Aa2 to increase its specific activity towards *Lygus* spp. and the subsequent identification of the transgenic event GH_A710504 designated MON88702 that has been advanced for development of commercial cotton varieties for *Lygus* control.

## Results

### Cry51Aa2 structure

To help guide protein optimization efforts, the crystal structure of Cry51Aa2 was solved to 2.3-Å resolution (Supplementary Table 1), using two crystals and the coordinates deposited in the PDB database (5HD2). Crystal 1 was used to solve the structure by SeMet multi-wavelength anomalous dispersion (MAD) phasing methods. Crystal 2 was used to solve the X-ray structure to higher resolution. A stereo image of a portion of the electron density map for crystal 2 is presented in Supplementary Fig. 1. Cry51Aa2 protein crystallizes in tetragonal crystal lattices (*a*,*b*=55–56 Å, *c*=209–210 Å, all angles 90°) and in the P43212 space group. The structure reveals an elongated predominantly β-stranded structure with similarities to other bacterial β pore-forming proteins. Recently, the structure of the closely related Cry51Aa1 (1.65 Å) protein was published^13^. The structure of Cry51Aa2 closely resembles that of Cry51Aa1 (Supplementary Fig. 2), which is expected as these two proteins have high sequence homology (97.7% identity). A super-positioning alignment of Cry51Aa1 onto the SeMet-Cry51Aa2-L11M structure coordinates conducted using the align program in PyMOL revealed that the structures have a pair-wise root mean square difference in α-carbon positions of 0.57 Å (using 291 α-carbon pairs). Also, Cry51Aa1 and Cry51Aa2 proteins crystallize in nearly identical tetragonal crystal lattices and in the identical space group.

### Cry51Aa2 variant proteins with improved activity

The project to improve the insecticidal activity of the Cry51Aa2 protein by mutagenesis and bioassay screening was initiated before the availability of a Cry51Aa crystal structure. As a first step, every residue in Cry51Aa2 was mutated to alanine to identify positions critical for protein function and to identify improved variants. In addition to single alanine scans, double alanine variants (residues 1+3, 2+4, 3+5 and so on) were also generated. Positions already containing alanine residues were changed to serine. The collection of variants expressed and tested covered 283 of the 308 positions within the amino-acid sequence. Insect bioassays were initially conducted only with *L. hesperus* due to the unavailability of a robust *L. lineolaris* colony to support a large-scale screening effort. From an initial pool of 54 putative hits, a total of 15 variants from the alanine and double alanine scans were confirmed to exhibit increased activity towards *L. hesperus* nymphs when compared with the native Cry51Aa2 protein (Supplementary Table 2). The most active variants showed three- to fourfold higher activity compared with the native Cry51Aa2 protein in a concentration response assay with an estimated LC_50_ of ∼12–20 p.p.m. (Supplementary Fig. 3). These variants included E125A, F147A and Q149A, and the double variants T145A-F147A, F147A-Q149A and Q149A-S151A. Subsequent combinatorial mutagenesis identified several other variants with significant improvements in activity compared with the native Cry51Aa2 protein (Supplementary Table 2).

Comparisons with related sequences also revealed key differences between Cry51Aa2 and Cry51Aa1; notably, the Cry51Aa1 sequence includes a deletion of three contiguous residues (HYS) in residue range 196–201 and four single amino-acid substitutions (F46S, Y54H, S167R and S217N; Supplementary Fig. 4). Incorporating some of these deletions or substitutions in Cry51Aa2 resulted in a significant improvement in activity against *L. hesperus.* For example, variant Cry51Aa2.807_5, containing a HYS motif deletion in residues 196–201, exhibited a threefold improved activity against *L. hesperus*, while variant Cry51Aa2.834, containing the HYS deletion as well as all four amino-acid substitutions in Cry51Aa1, exhibited a 12-fold improved activity (Table 1). Guided by these results and the Cry51Aa2 structure, Cry51Aa2.834 alanine scan variants surveying 53 surface-exposed residues were tested in the *L. hesperus* assay with several variants showing increased activity compared with Cry51Aa2.834, notably variants with alanine substitutions at positions 93, 95, 147 and 149 (Table 1; Supplementary Fig. 5). Subsequent combinatorial and saturation mutagenesis at these positions led to the identification of Cry51Aa2.834 variants with significant improvements in activity towards *L. hesperus*, including Cry51Aa2.834_5 (S95A, F147A), Cry51Aa2.834_6 (T93A, F147A) and Cry51Aa2.834_7 (Q149E) (Table 1). The LC_50_ for the best variant, Cry51Aa2.834_5, was 90-fold lower than that of the original Cry51Aa2 protein.

The variants identified as good candidates in *L. hesperus* bioassays did not show improved activity on *L. lineolaris*. Most notably, Cry51Aa2.834_5 was only marginally more active than Cry51Aa2 against *L*. *lineolaris*. To address this challenge, Cry51Aa2.834_5 was selected as a template for further mutagenesis, and variants were screened against both *L. hesperus* and *L. lineolaris* to identify those variants with improved activity against both species. Computational analysis of the Cry51Aa2 structure indicated that the head region near the engineered deletion (residue range 196–201) and the hypervariable sequence block defined by the related crystal protein TIC853 (residues 210–221; Supplementary Fig. 4; ref. 14) were likely involved in protein–protein interactions^15^ that could impact oligomeric assembly, pore formation efficiency or species specificity through insect–receptor interactions. Therefore, increased sampling of modifications in these regions was performed with substitutions expected to be more likely involved in protein–protein or protein–sugar interactions (see Methods). In addition, amino-acid positions showing variation among Cry51Aa1, Cry51Aa2 and TIC853 proteins (Supplementary Fig. 4) were targeted for mutagenesis in Cry51Aa2.834_5, substituting amino acids found at those positions in the other similar proteins. These approaches identified Cry51Aa2.834_13, consisting of a reversion of the S217N mutation and the introduction of a P219R mutation, significantly improving activity against *L. lineolaris* but reducing activity against *L. hesperus* (Table 1). Further mutagenesis of Cry51Aa2.834_13 comprised a hit-on-hit approach in which other favourable amino-acid substitutions identified through screening of Cry51Aa2.834 or Cry51Aa2.834_5 variants were introduced and tested on both *Lygus* species to identify promising combinatorial variants. Several of these variants, (Cry51Aa2.834_14 — Cry51Aa2.834_16) exhibited marked increases in *L. lineolaris* activity while retaining, and even improving, efficacy towards *L. hesperus* (Table 1). The highest increase in activity against *L. lineolaris* (262-fold) was observed between Cry51Aa2.834_5 and Cry51Aa2.834_16. Cry51Aa2.834_16 also exhibits about threefold higher toxicity against *L. hesperus* (LC_50_=0.3 μg ml^−1^) than against *L. lineolaris* (LC_50_=0.85 3 μg ml^−1^). Accordingly, a synthetic coding region and expression cassette (Supplementary Fig. 6) was designed for Cry51Aa2.834_16 to enable expression in cotton plants and an evaluation of *in planta* efficacy towards *Lygus* species. Following cotton transformation, R2 seeds homozygous for a single copy of the Cry51Aa2.834_16 expression cassette were selected for propagation and advanced testing.

### Protein accumulation in plants

Cry51Aa2.834_16 accumulation was significantly different among transgenic events (*F*=11.17, d.f.=3,203, *P*<0.0001, least square means (LSMEANS) test, *n*=8), sampling time (*F*=3.75, d.f.=2,203, *P*=0.0253, LSMEANS test, *n*=8) and tissues (*F*=89.91, d.f.=2,203, *P*<0.001, LSMEANS test, *n*=8) but not among locations (Table 2). Further, there was a significant interaction between sampling time and transgenic events (*F*=2.3, d.f.=6,203, *P*=0.0361, LSMEANS test, *n*=8), but not between sampling time and tissue, transgenic event and tissue, and between sampling time, tissue and transgenic events. Accumulation of Cry51Aa2.834_16 was highest in squares followed by bolls and leaves. Protein accumulation did not change over time except for event GH_A710504, which exhibited reduced Cry51Aa2.834_16 accumulation in square tissue at 90 days after planting (DAP). Protein accumulation in leaves was the lowest among tissue types in all transgenic events.

### Field insect bioassays

**Figure 1:**
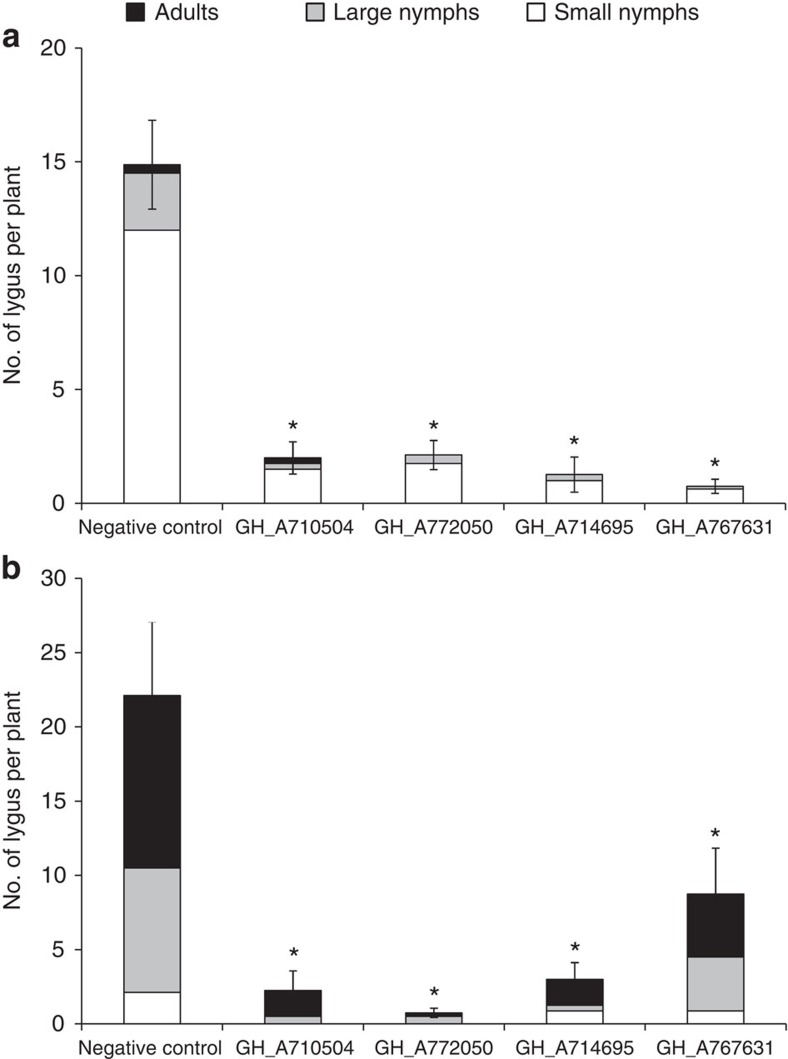
Efficacy of transgenic cotton plants on *Lygus* spp. Total number of Lygus and their developmental stages on transgenic cotton plants expressing Cry51Aa2.834_16 on *L*. *lineolaris* (**a**) and *L*. *hesperus* (**b**) in the field whole-plant caging experiments. The error bars represent s.e.m. for total numbers of *Lygus*. * indicates a significant difference between the transgenic event and the negative control at *P*=0.05 (LSMEANS test, *n*=8).

All four Cry51Aa2.834_16 events were efficacious in reducing numbers of *L*. *lineolaris* (Fig. 1a) and *L*. *hesperus* (Fig. 1b) when compared with the negative control (*P*<0.05, LSMEANS test, *n*=8). Seven- to 19-fold fewer *L. lineolaris* were recorded on transgenic events compared with the negative control. In addition, 10-fold fewer nymphs and 63-fold fewer adults were recorded from these events compared with the negative control. For *L. hesperus*, total number of insects was reduced by 3–30-fold on transgenic events. On average, 0.25–4.25 adults were recovered from these transgenic events, whereas 11.63 adults were recovered from the negative control.

## Discussion

Crystal proteins from *Bt* belonging to the insecticidal three-domain Cry toxin family have long been the subject of both protein improvement efforts and structure–function studies^16^,^17^,^18^,^19^. Another major class of Cry proteins belonging to the β pore-forming bacterial toxin family has received far less attention. Previously, we described the isolation of one such crystal protein, designated Cry51Aa2, with activity towards the hemipteran pests *L. hesperus* and *L. lineolaris*^8^. Here we report the modifications made to this protein to increase its specific activity towards *Lygus* spp. and to enable the development of transgenic cotton event MON88702 for *Lygus* control.

**Figure 2:**
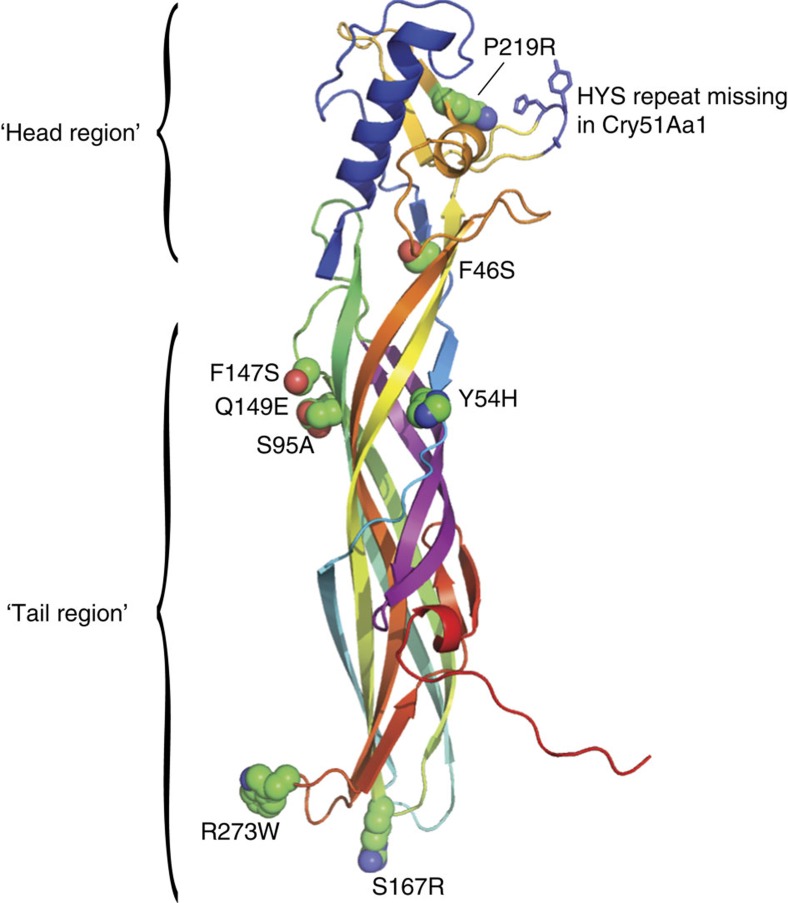
The Cry51Aa2 crystal structure. Cry51Aa2 structure (2.28-Å resolution) with the Cry51Aa2.834_16 side-chain substitutions rendered in space-filling sphere mode. Ribbon structure is coloured from N to C terminus in rainbow mode from blue to red. The HYS repeat missing in Cry51Aa2.834_16, which is present in Cry51Aa2 but not in Cry51Aa1, is in dark-blue-stick representation. The putative β-pore-forming hairpin loop (residues 112–140) is coloured magenta and is at the midsection of the structure.

The structure of the Cry51Aa2 protein displaying the amino-acid substitutions found in the optimized protein Cry51Aa2.834_16 is shown in Fig. 2. Key amino-acid substitutions with differential effects on activity towards *L. hesperus* and *L. lineolaris* reside in the ‘head region' of the molecule, also referred to as ‘domain 1' (ref. 13), supporting the hypothesis that this domain is involved in determining specificity. This domain comprises two distinct segments spanning residues 2–43 and 195–233 (Supplementary Fig. 4). Substitutions at positions 217 and 219 in variant Cry51Aa2.834_5 caused a 27-fold increase in activity towards *L. lineolaris* but a 12-fold decrease in the activity towards *L. hesperus* (Table 1). Deletion of a single HYS motif in the loop region comprising positions 196–201, also within this domain, improved Cry51Aa2 activity towards *L. hesperus* by threefold but a side-by-side comparison with Cry51Aa2 showed no improved activity towards *L. lineolaris* (Supplementary Fig. 7). Similarly, Cry51Aa2.834 was indistinguishable from Cry51Aa2 in *L. lineolaris* assays despite containing the same HYS deletion. Interestingly, substitutions at position 224 in the head region of the related β pore-forming toxin Mtx-2 of *Lysinibacillus sphaericus* impacts the specificity of this protein towards *Culex quinquefasciatus* and *Aedes aegypti* mosquito larvae: a K224T substitution abolishes activity towards *Culex* larvae, but increases activity towards *Aedes* larvae ∼100-fold (ref. 20). Substitutions at positions 93, 95, 147, 149 and 251 reside on adjacent antiparallel β-strands in the region adjacent to the putative amphipathic pore-forming loop (residues 112–140) and define a second region within the Cry51Aa2 structure impacting insecticidal activity (Supplementary Fig. 8). While it is not clear why the specific mutations at these five positions led to improvements in insecticidal activity, they were each identified by alanine scan mutagenesis and all involve surface-exposed side chains. Surface-exposed side-chain substitutions in residues engaged in antiparallel β-sheets, as is the case here, should not impact the lateral hydrogen bonds which stabilize the β-sheet, but could impact other processes such as protein oligomerization or pore formation. Finally, the R273W substitution resulting in increased *Lygus* activity (for example, Cry51Aa2.834_15 in Table 1) is located in the tail region of the molecule (Fig. 2) and may promote interactions with the target cell membrane due to increased hydrophobicity at that position.

Preliminary studies using neonate *Lygus* to infest plants indicated low survivorship of insects in small-cage experiments^8^. Whole-plant caging with adults has proven to be a robust method for *Lygus* efficacy evaluation and has more field relevance as cotton is typically infested by adults from neighbouring vegetation^7^. In addition, we ensured that the females released were sexually mature and most likely premated, and ready to oviposit when released on cotton plants. Infestation of two males and two females per plant increased the chances of successful mating and continuous oviposition, and created high insect pressure for a thorough evaluation of efficacy. Allowing infested plants and insects to grow for 1 month was a sufficient time for *Lygus* eggs to hatch and develop to next-generation adults^7^. Although the number of eggs laid per plant was unknown, the total number of next-generation insects recovered per plant was a good indicator of efficacy.

Differential efficacy of transgenic events could partially be attributed to protein accumulation differences, although correlation analysis yielded variable relationships across tissues and sampling time. The lack of a consistent relationship could be due to the differences in locations where both studies were conducted, protein expression, protein stability in cotton tissues or other unknown factors. The level of efficacy observed with Cry51Aa2.834_16 should provide economic control of *Lygus* bugs in cotton. On the basis of these results and further characterization (field efficacy, agronomics and molecular), cotton event GH_A710504 was advanced for further development as a *Lygus* control product and designated as MON88702. Results presented here demonstrate an important development in management options for *Lygus* spp., which have developed resistance against several classes of insecticides^21^. Currently, chemical insecticides are the only option available and multiple applications are used throughout the growing season. A transgenic option for *Lygus* management using MON88702 could reduce or eliminate sprays required for *Lygus* control and lessen the environmental impact of chemical insecticide use on cotton while preserving yield.

## Methods

### Structure determination of Cry51Aa2

The initial X-ray crystal structure of Cry51Aa2 was obtained by crystallizing the SeMet-Cry51Aa2-L11M protein construct and solving the structure via SeMet MAD phasing methods to 2.75-Å resolution. The structure was later extended to 2.28-Å resolution using a higher-resolution data set. Data collection, phasing and refinement statistics for the initial 2.75-Å resolution structure (crystal 1) and the later 2.28-Å resolution structure (crystal 2) are listed in Supplementary Table 1.

*E. coli*-expressed SeMet-Cry51Aa2-L11M protein construct crystals were produced using vapour diffusion by the sitting-drop protein crystallization method^22^. The initial protein solution was 5.5 mg ml^−1^ SeMet-Cry51Aa2-L11M in 25 mM sodium carbonate buffer (pH 10.5). The reservoir solution was 500 μl of 2.0 M sodium chloride and 50 mM HEPES buffer (pH 7.5). Diamond-shaped crystals resulted from 2-μl drops, using 0.7 μl protein solution and 1.3 μl well solution.

Crystals were prepared for low-temperature (∼100 K) X-ray intensity data collection by dipping in a cryosolution for ∼5–10 s before plunging in liquid nitrogen and capping in cryovials. The cryosolution was 2.2 M sodium chloride, 50 mM HEPES buffer (pH 7.5) and contained 25 (w/v)% of the cryoprotectant ethylene glycol. X-ray diffraction data were collected at the Southeast Regional Collaborative Access Team (SER-CAT) 22-BM beamline, which is in the Advanced Photon Source synchrotron at Argonne National Labs. Four wavelengths of MAD X-ray diffraction data (*λ*_1_=0.97972 Å, *λ*_2_=0.97228 Å, *λ*_3_=0.97996 Å and *λ*_4_=0.98400 Å) were collected about the selenium absorption edge of crystals. Crystal diffraction analyses revealed that Cry51Aa2 belonged to the tetragonal crystal system, space group P4_3_2_1_2 (space group #96) or its enantiomorph, space group P4_1_2_1_2 (space group #92), and possessed unit cell lengths of *a*=*b*=55.4 Å, *c*=208.3 Å, with all angles 90°. All data sets were obtained using a Marresearch mar225 charge-coupled device detector, and each data set contained 300° of data collected using 6-s exposures, an oscillation angle of 1° and crystal-to-detector distance of 250 mm. Data were processed using the HKL2000 package^23^.

The SOLVE/RESOLVE program^24^,^25^, version 2.08, was used to locate selenium sites, calculate phases and obtain an initial peptide trace of the structure from the peak (*λ*_1_=0.97972 Å), high-energy remote (*λ*_2_=0.97228 Å) and inflection (*λ*_3_=0.97996 Å) data sets using 20–2.8 Å data. Phasing work revealed that the crystal true space group is P4_3_2_1_2 (#96). Electron density maps were calculated using CCP4 (Collaborative Computational Project, Number 4, 1994), and map evaluation and structure model building to the maps conducted using program O (ref. 26) on a linux workstation with a stereographics monitor. The SeMet-Cry51Aa2-L11M protein construct contains seven Met residues. Map inspection of phasing results located all Se sites (except for the N-terminal SeMet residue), and the electron density map had clear side-chain definition, allowing all six SeMet residues to be properly assigned to true sequence. Using the InsightII Biopolymer module (Accelrys, Inc.), true sequence peptides were generated for map-fitting in program O (ref. 26). Eventually, all 309 construct residues were fit to the electron density map. Crystallographic refinement was conducted with the CNX2002 program (Accelrys, Inc.) using peak data over the resolution range 20–2.8 Å. Structurally bound water molecules were added in the latter stage of the 2.8-Å resolution refinement. This structure was later refined with the refmac5 crystallographic refinement program^27^, as implemented in the CCP4 program suite^28^ using 53–2.75-Å peak wavelength data. The resulting structure has a working R-factor=21.8% (using 95% of the data set), and a free R-factor=30.4% (using 5% of the data set); it contains 308 residues, lacking only the N-terminal methionine and 66 water molecules (Supplementary Table 1).

This SeMet-Cry51Aa2-L11M structure was extended to 2.28-Å resolution using a 2.28-Å resolution data set collected at the SER-CAT 22-ID beamline (at a wavelength of *λ*=1.000 Å). Data were obtained using a Marresearch mar300 charge-coupled device detector, and 115 data frames were collected using 3-s exposures, an oscillation angle of 1° and crystal-to-detector distance of 200 mm. Data were processed using the HKL2000 package^23^. The Phaser crystallographic software package^29^ was used to position the 2.8-Å resolution protein structure in the new data set cell. The higher-resolution structure was refined using the refmac5 program^25^ in CCP4 (ref. 25). This higher-resolution SeMet-Cry51Aa2-L11M protein structure contains all but the N-terminal Met residues in the crystallization construct, 46 water molecules and has a working R-factor=23.8% (uses 95% of the data set) and a free R-factor=29.5% (using 5% of the data set) using 53.7–2.28-Å resolution data (Supplementary Table 1). Cry51Aa2 atomic structure coordinates are deposited with the PDB (5HD2).

### Mutagenesis strategies

Semi-random mutagenesis, homologue scanning based on multiple sequence alignments with related Cry51 proteins, and predictive structure design approaches were used to improve the activity of Cry51Aa2. Every residue in Cry51Aa2 was mutated to alanine to identify positions critical to protein function and to identify improved variants. Positions already containing alanine residues were mutated to serine. In addition to single alanine mutants, double alanine mutants (residues 1+3, 2+4, 3+5 and so on) were also generated. Positions subjected to saturation mutagenesis in Cry51Aa2 included Q70, S95, E125, G128, I134, E135, N137, A139, T145, F147, Q149, A150, S151, D153, W156, I158, S159, V175, T182, L187, H199, I306 and T308. Mutations in the Cry51Aa2 putative pore-forming loop region (positions 115–138) were created by replacing alternating hydrophilic amino acids with other hydrophilic amino acids and similarly for the hydrophobic amino acids. To reduce the number of variants tested in insect bioassay, partial alanine scanning mutagenesis was performed on Cry51Aa2.834, targeting only those positions where the residue solvent accessible surface area was >15%. Those positions included T68, Q70, A87, T93, S95, G105, S117, S119, E125, G128, I134, A139, T145, F147, Q148, Q149, A150, S151, D153, E155, W156, I158, S159, R167, V175, M177, T182, L187, H196, Y197, S198, W205, Y221, F232, D238, T240, T242, T244, S246, V247, S249, T271, I272, R279, H284, S290, N292, E296, M297, T300, P302, I303 and T305. As before, alanines at position 87, 139 and 150 were changed to serine. Note that for all of the Cry51Aa2.834 variants, amino-acid positions after position 198 are shifted relative Cry51Aa2 due to the single HYS deletion between positions 196 and 201. On the basis the bioassay results from the alanine scan, the following positions in Cry51Aa2.834 were subjected to saturation mutagenesis: E125, T133, I134, E135, N137, F147, Q149, A150, E155 and N157. Homologue scanning mutagenesis relied on alignment of the Cry51Aa1, Cry51Aa2 and TIC853 (ref. 14) amino-acid sequences to identify positions of variability (Supplementary Fig. 4). Variant positions were targeted for mutagenesis in Cry51Aa2.834_5 with the aim of identifying naturally occurring substitutions that impact activity. Other improvement strategies relied on the determined atomic structure of Cry51Aa2. Prediction of potential protein–protein interaction sites was determined using Molsoft ICM-pro^13^. Generally, these substitutions were confined to the ‘head' region of the protein and selected positions were mutated to aromatic or hydrogen-bonding groups to favour binding to sugar groups found on glycosylated receptors^30^,^31^. These substitutions in Cry51Aa2.834_5 included N15Y, W17H, W17Y, P19Y, N21R, N21Y, N21W, G24S, G24Y, I25Y, Q26K, Q26Y, D40W, D40Y, S42Y, I43N, I43R, I43S, I43W, I43Y, T45Y, I52H, E195Y, H196N, H196S, Y197W, Y197S_S198Y, Y197F_S198N, S198N, Y200N, Y200S, P201G, I202Y, W205H, S207H, S207N, S207R, S207W, S207Y, S208H, S208R, S208W, S208Y, D210K, D210N, S212N, S212Y, Y213W, N214V, N214W, N214Y, P216N, P216S, P216W, P216Y, P216A_M218Y, M218F, M218I, M218K, M218L, M218Y, W220H, W220S, W220Y, W220Y_Y221W, Y221F, Y221W, N224T, N224W, N224Y, L235F, L235N, L235T, L235Q, L235Y and N239Q. Additional mutagenesis strategies included those aimed at impacting proteolytic activation or membrane interaction, for example, introducing aromatic amino acids to favour interactions with membranes. An analysis of the alpha-hemolysin structure (7AHL.pdb) indicates that positions likely to interact with the membrane correspond to residues in the Cry51Aa2 tail region. Several positions in the Cry51Aa2 tail region were selected for mutation to F, Y or W including L78, I123, H270, R273 and I275.

### Cry51Aa2 variant construction and protein production

Mutagenic oligonucleotides (Integrated DNA Technologies, IDT, Coralville, Iowa) were designed as sense and antisense pairs with a 15–20 bp overlap, and no significant secondary structure at the 3′ ends, to enable whole-plasmid PCR (Supplementary Data 1). Plasmid pMON106128 served as the base vector for the Cry51Aa2 and Cry51Aa2.834 coding region templates (Supplementary Data 2). Variant clones generated by site-directed mutagenesis were confirmed by sequencing and transformed into *B. thuringiensis* using electroporation. Expressed crystal proteins recovered from sporulated and lysed cultures were solubilized in 25 mM sodium carbonate (pH 10.5) buffer, analysed by SDS–PAGE to confirm the presence of a single band, and quantified by densitometry using bovine serum albumin as a standard^8^. For high-throughput screening of variant proteins, crystals were solubilized in carbonate buffer and supernatant total protein was quantified using the modified Bradford protein assay kit from Bio-Rad Laboratories (Hercules, CA). Total protein recovery from a *B. thuringiensis* strain containing the base vector pMON106128 was used to establish background protein concentration in test samples.

### Insect sources and diet bioassays

*L. hesperus* and *L. lineolaris* eggs were obtained from the laboratory culture at National Biological Control Laboratory, USDA-ARS (Stoneville, MS). A 96 well micro-titre plate artificial diet bioassay was used to evaluate protein variant activity^8^. Briefly, diet and toxin samples were mixed at equal proportions and packaged into parafilm sachets. Insects were allowed to feed for 5 days at 27 °C, 60% relative humidity (RH) and a 14:10 (light:dark) h photoperiod. Bioassay capacity for *L. lineolaris* was limited compared with *L. hesperus*, so screening was performed initially against *L. hesperus* and improved variants were further modified and screened against *L. lineolaris*. Variants were screened at a single concentration sufficient to detect activity improvements and putative hits confirmed by testing at multiple concentrations. Select variants were advanced for further testing to determine LC_50_ values to provide a quantitative measure of improvement. Assays included 8–10 protein concentrations with sample buffer as untreated control. Twenty-four insects were tested per concentration and assays replicated twice.

### Cotton transformation

Synthetic coding regions for 13 different Cry51Aa2 variants were designed using 29 constructs (up to seven constructs per variant) and transformed into cotton to confirm that improved activity observed in artificial diet bioassays translate to improved activity *in planta* (data not shown). Similarly, the synthetic coding region for Cry51Aa2.834_16 was designed to enable efficient cotton expression^32^. The expression cassette in pMON139006 contains the 35S promoter from the figwort mosaic virus, untranslated leader sequence from *Hsp*81 of *Arabidopsis thaliana* (nt 1–1,490), *Cry51Aa2.834_16* coding region (nt 1,499–2,419) and the cauliflower mosaic virus 35S terminator (nt 2,426–2,625; Supplementary Fig. 6). Transformed cotton plants were generated following the protocol outlined by Chen *et al*.^33^ using *DP393* as the transformation germplasm. Bolls were collected and seeds (R1 generation) were stored at 10±1 ° C, 50% RH, 24-h dark. Seeds were planted in the greenhouse, plants were tested for gene zygosity using a DNA assay and R2 generation seeds were collected only from homozygous plants.

### Protein accumulation in plants

Cry51Aa2.834_16 accumulation was measured from two field trials in Chenyville, LA, and Winterwille, MS. Seeds from four transgenic events and *DP393* were planted in eight 30-ft rows and grown as per the cotton growing recommendations. Tissues were collected from 10 randomly selected plants in each plot at ∼45 (terminal leaf and square), 70 (terminal leaf, square and boll) and 90 (terminal leaf, square and boll) DAP, which represented peak squaring, peak bloom and peak boll developmental stages, respectively. Tissues were placed on dry ice and shipped to the laboratory for additional processing. Bracts and lint were removed from squares and bolls, respectively, before lyophilization. Protein accumulation in lyophilized tissues was measured using an enzyme-linked immunosorbent assay^8^. Cry51Aa2 polyclonal antibodies were used to measure Cry51Aa2.834_16 accumulation in plant tissues.

### Field insect bioassays

Field trials were conducted in Casa Grande, AZ, and Jerseyville, IL, for *L*. *hesperus* and *L*. *lineolaris*, respectively. R3 generation seeds were sown in two 15-ft row plots for four transgenic events expressing *Cry51Aa2.834_16* with *DP393* used as a negative control. At 15–20 DAP, plants were thinned to achieve uniform stand. At 35–40 DAP, individual plants were carefully examined to remove any pests and predators present and enclosed with 150 × 228-cm white solid voile cages (JoAnne Fabrics, item number 8139875).

*Lygus* nymphs (predominantly third instars and above) were collected from alfalfa or canola fields and laboratory reared for up to 2 weeks using organic green beans or snap peas. At eclosion, adults were collected and moved to new containers to assess their reproductive maturity. At least 100–150 adults (∼1:1 sex ratio) were placed in plastic containers (19.2 Gal, Rubbermaid) for successful mating. At infest all adults were at least 6 days old and cotton plants were 45–50 days old (peak squaring stage). Eight plants per event were infested by releasing two pairs of male and female adults into cages. After 30 days, plants were cut below the cage and moved to the laboratory to collect and count insects. Numbers of next-generation insects and their developmental stage were recorded for each plant. Nymphs ≤3rd and ≥4th instar were recorded as small and large nymphs, respectively.

### Statistical analyses

Protein variant activity on both *Lygus* species was first examined for variance homogeneity using Levene's test^34^. If the variance was homogeneous, then mean percentage mortality was compared with effects in the untreated control using Dunnett's test (*α*=0.05)^35^,^36^. LC_50_ values for the activity of improved variants on both *Lygus* species were calculated using probit analysis^37^,^38^.

Protein accumulation in multiple tissues was analysed for analysis of variance using PROC MIXED and means separated by LSMEANS test (*α*=0.05). Analyses of whole-plant caging experimental results were conducted separately for each insect species. Total number of next-generation *Lygus* recovered from whole-plant caging studies were analysed using PROC MIXED and means separated by the LSMEANS test (*α*=0.05).

### Data availability

Crystallography data referenced in this study are available in PDB with the accession code 5HD2. The authors declare that all other data supporting the findings of this study are available within the article and its Supplementary Information files or from the corresponding author upon request.

## Additional information

**How to cite this article:** Gowda, A. *et al*. A transgenic approach for controlling *Lygus* in cotton. *Nat. Commun.* 7:12213 doi: 10.1038/ncomms12213 (2016).

## Supplementary Material

Supplementary Information Supplementary Figures 1-8 and Supplementary Tables 1-2

Supplementary Data 1 Mutagenic oligonucleotide sequences

Supplementary Data 2 Coding regions for Cry51Aa2 and Cry51Aa2.834

## Figures and Tables

**Table 1:** Activity of Cry51Aa2 protein variants on *L*. *hesperus* and *L*. *lineolaris* (LC_50_ values in μg protein per ml).

Protein	Amino-acid difference(s) relative to Cry51Aa2	*L. hesperus*				*L. lineolaris*			
		LC_50_	95% CI	Slope±s.e.	Chi-square	LC_50_	95% CI	Slope±s.e.	Chi-square
Cry51Aa2	Parent	73	50.2–116.35	1.77±0.27	43.28	>223	Not estimated		
Cry51Aa2.807_5	HYS_ Δ(196–201)	22.4	14.8–39.9	1.93±0.35	29.48	ND			
Cry51Aa2.834	F46S, Y54H, S167R, S217N, HYS_ Δ(196–201)	5.9	3.95–9.35	1.52±0.21	52.86	>223	Not estimated		
Cry51Aa2.834_2	F46S, Y54H, T93A, S167R, S217N, HYS_ Δ(196–201)	2.9	2.25–3.85	2.12±0.25	72.22	ND			
Cry51Aa2.834_3	F46S, Y54H, S95A, S167R, S217N, HYS_ Δ(196–201)	2.4	1.15–5.8	1.94±0.47	17.16	ND			
Cry51Aa2.834_4	F46S, Y54H, F147A, S167R, S217N, HYS_ Δ(196–201)	1.1	0.7–1.7	1.30±0.16	64.43	ND			
Cry51Aa2.834_6	F46S, Y54H, T93A, F147A, S167R, S217N, HYS_ Δ(196–201)	1.45	1.0–2.15	2.06±0.30	46.28	ND			
Cry51Aa2.834_7	F46S, Y54H, Q149E, S167R, S217N, HYS_ Δ(196–201)	1.4	0.87–2.34	1.77±0.29	37.54	ND			
Cry51Aa2.834_5	F46S, Y54H, S95A, F147A, S167R, S217N, HYS_ Δ(196–201)	0.8	0.6–1.15	1.82±0.21	72.08	223	153.5–314	1.84±0.55	11.15
Cry51Aa2.834_13	F46S, Y54H, S95A, F147A, S167R, P219R, HYS_ Δ(196–201)	9.9	4.45–10.15	1.03±0.10	105.38	8.3	7.85–9.35	3.16±0.57	31.27
Cry51Aa2.834_14	F46S, Y54H, S95A, F147A, S167R, P219R, V251A, HYS_ Δ(196–201)	0.6	0.1–1.6	1.01±0.26	14.81	4.8	3.05–6.55	2.98±0.38	60.02
Cry51Aa2.834_15	F46S, Y54H, S95A, F147A, S167R, P219R, R273W, HYS_ Δ(196–201)	1.35	0.95–1.93	1.75±0.23	59.35	5.9	5.0–6.6	2.04±0.35	34.7
Cry51Aa2.834_16	F46S, Y54H, S95A, F147S, Q149E, S167R, P219R, R273W, HYS_ Δ(196–201)	0.3	0.05–0.75	0.94±0.26	12.86	0.85	0.5–1.5	1.07±0.15	48.65
Cry51Aa2.834_17	F46S, Y54H, S95A, F147A, S167R, P219R, N239A, V251A, HYS_ Δ(196–201)	0.4	0.15–0.8	0.94±0.14	44.43	1.2	0.85–1.7	0.95±0.10	82.24

CI, confidence interval; ND, not determined.

HYS_Δ196–201=contiguous HYS deletion in residue range 196–201, *n*=40 (replications=4–5).

**Table 2:** Cry51Aa2.834_16 accumulation (μg mg^−1^ dry weight) in various tissues of transgenic cotton plants.

Sampling time (DAP)	Tissue	Transgenic events			
		GH_A710504	GH_A772050	GH_A714695	GH_A767631
45	Leaf	505.68±79.36 a A	519.98±46.50 a A	381.89±27.09 a A	321.02±36.75 a A
	Square	935.31±115.04 ab B	1164.21±98.09 b A	854.43±79.40 a A	752.53±91.76 a A
70	Leaf	424.68±51.12 a A	398.21±58.56 a A	379.86±33.10 a A	292.69±15.07 a A
	Square	1070.63±85.83 b B	714.49±179.65 a A	859.93±167.21 ab A	641.59±52.43 a A
	Boll	450.41±26.25 a A	546.03±77.72 a A	392.48±55.36 a A	459.96±54.52 a A
90	Leaf	396.97±43.72 ab A	472.07±35.73 b A	365.14±28.39 ab A	246.04±15.53 a A
	Square	703.45± 139.79 ab A	1038.1±122.07 c A	804.14±119.61 bc A	506.74±74.59 a A
	Boll	498.99±65.54 ab A	706.36±104.87 b A	416.24±80.77 a A	497.78±110.28 ab A

DAP, days after planting.

Means followed by different lower-case letters within a row and different upper-case letters within a column are significantly different from each other at *P*≤0.05 (LSMEANS test, *n*=8). Data shown as mean±s.e.m.
