# Author Correction: A transgenic approach for controlling *Lygus* in cotton

**DOI:** 10.1038/s41467-020-14789-w

**Published:** 2020-02-26

**Authors:** Anilkumar Gowda, Timothy J. Rydel, Andrew M. Wollacott, Robert S. Brown, Waseem Akbar, Thomas L. Clark, Stanislaw Flasinski, Jeffrey R. Nageotte, Andrew C. Read, Xiaohong Shi, Brent J. Werner, Michael J. Pleau, James A. Baum

**Affiliations:** 1 Monsanto Company, Chesterfield, MO 63017 USA; 2 Present Address: Visterra Inc, Cambridge, Massachusetts, 02139 USA; 3 Present Address: Plant Pathology and Plant-Microbe Biology, School of Integrative Plant Science, Cornell University, 334 Plant Science, Ithaca, NY 14853 USA

Correction to: *Nature Communications* 10.1038/ncomms12213, published online 18 July 2016

The original version of this Article contains an error in the title of Table 2, which incorrectly states Cry51Aa2.834_16 accumulation in units of ‘µg mg^−1^ dry weight’ in place of ‘µg g^−1^ dry weight ’. The correct version of this Table appears below as Table 1.

**Table 2:** . Cry51Aa2.834_16 accumulation (µg g^−1^ dry weight) in various tissues of transgenic cotton plants.

Sampling time (DAP)	Tissue	Transgenic events			
		GH_A710504	GH_A772050	GH_A714695	GH_A767631
45	Leaf	505.68 ± 79.36 a A	519.98 ± 46.50 a A	381.89 ± 27.09 a A	321.02 ± 36.75 a A
	Square	935.31 ± 115.04 ab B	1164.21 ± 98.09 b A	854.43 ± 79.40 a A	752.53 ± 91.76 a A
70	Leaf	424.68 ± 51.12 a A	398.21 ± 58.56 a A	379.86 ± 33.10 a A	292.69 ± 15.07 a A
	Square	1070.63 ± 85.83 b B	714.49 ± 179.65 a A	859.93 ± 167.21 ab A	641.59 ± 52.43 a A
	Boll	450.41 ± 26.25 a A	546.03 ± 77.72 a A	392.48 ± 55.36 a A	459.96 ± 54.52 a A
90	Leaf	396.97 ± 43.72 ab A	472.07 ± 35.73 b A	365.14 ± 28.39 ab A	246.04 ± 15.53 a A
	Square	703.45± 139.79 ab A	1038.1 ± 122.07 c A	804.14 ± 119.61 bc A	506.74 ± 74.59 a A
	Boll	498.99 ± 65.54 ab A	706.36 ± 104.87 b A	416.24 ± 80.77 a A	497.78 ± 110.28 ab A

*DAP* days after planting.

Means followed by different lower case letters within a row and different uppercase letters within a column are significantly different from each other at *P* ≤ 0.05 (LSMEANS test, *n* = 8). Data shown as mean ± s.e.m.

