# Supplementary material for: A transgenic approach for controlling Lygus in cotton
Source: Nat Commun. 2016 Jul 18;7:12213. doi: 10.1038/ncomms12213 (PMC4960306; doi:10.1038/ncomms12213)
Supplement: Supplementary Information — Supplementary Figures 1-8 and Supplementary Tables 1-2 [file ncomms12213-s1.pdf]

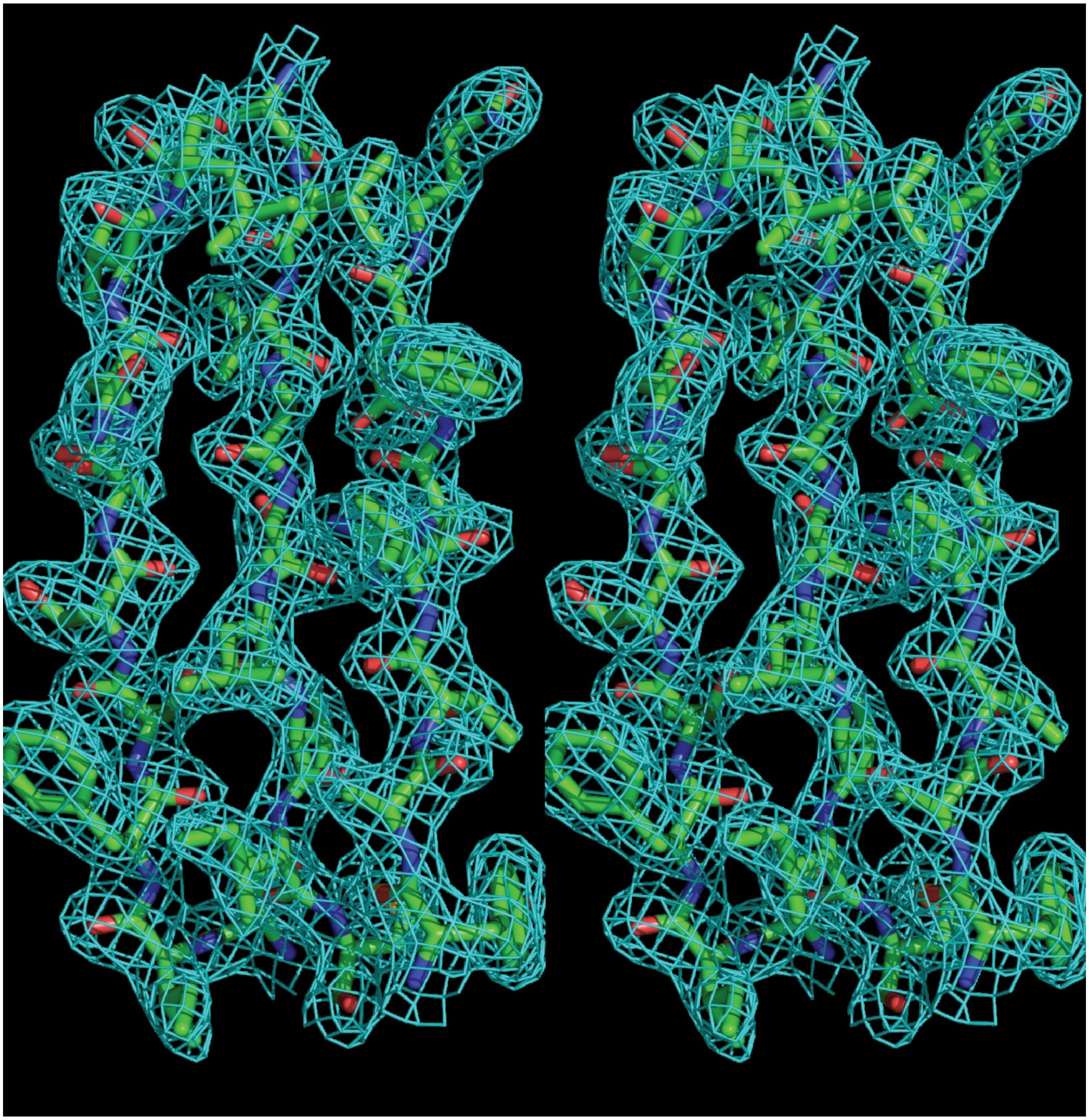

Supplementary Fig. 1. Stereo image of β–sheet structure and density map in Cry51Aa2: Residues displayed from left to right are 65-72, 169-177, and 259-266. The map is a 2Fo-Fc map contoured at 1 σ level

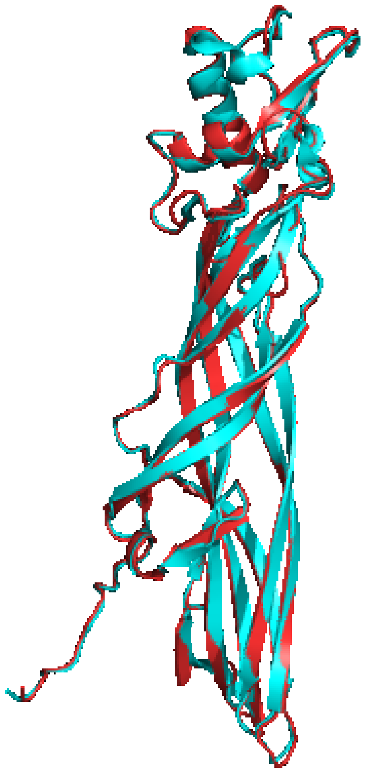

Supplementary Fig. 2. Cry51Aa1 (ref. 13) and Cry51Aa2 display a high level of structural similarity. Cry51Aa1 (1.65 Å resolution; cyan); Cry51Aa2 (2.28 Å resolution; red); r.m.s.d.=0.57 Å for 291 alpha carbon pairs.

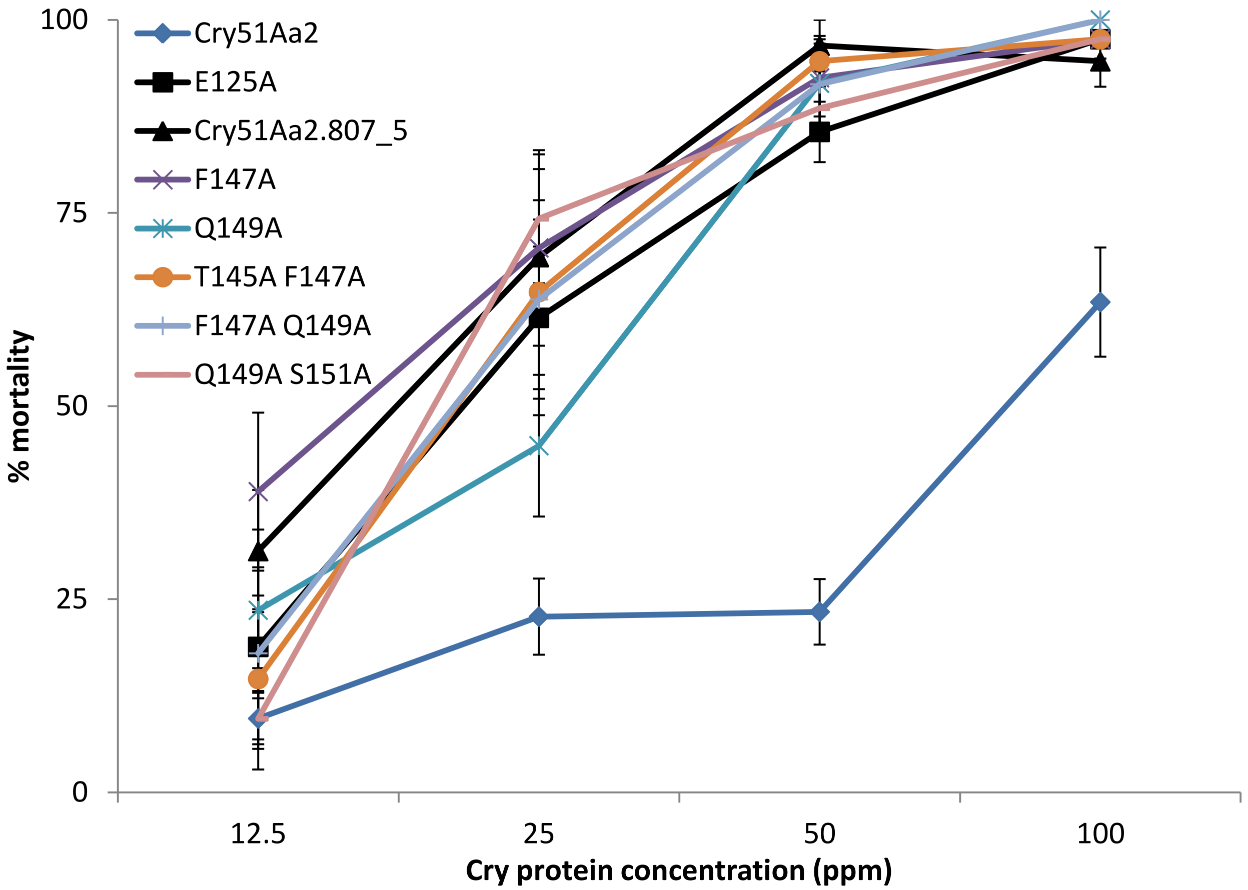

Supplementary Fig. 3. Concentration-response assay demonstrating improved activity of select Cry51Aa2 variants towards *L. hesperus.* Data shown as mean ± s.e.m. (n=24)

```
TIC853    MAILDLKSLV LDAINYWGPK NNNGIQGYNF NYPISERQID TSIITSTHSR LMPHDLTIPQ  60
Cry51Aa1  LAILDLKSLV LNAINYWGPK NNNGIQGGDF GYPISEKQID TSIITSTHPR LIPHDLTIPQ  63
Cry51Aa2  MAILDLKSLV LNAINYWGPK NNNGIQGGDF GYPISEKQID TSIITFTHPR LIPYDLTIPQ  60
          *           *                **  *     *         *  *   * *        60

TIC853    NLETIFTTTQ VLTNNTDVQQ SQTVSFSKKT TTTTSTSTTD GWTEGGRISD TLEENVSVSI 120
Cry51Aa1  NLETIFTTTQ VLTNNTDLQQ SQTVSFAKKT TTTTSTSTTN GWTEGGKISD TLEEKVSVSI 123
Cry51Aa2  NLETIFTTTQ VLTNNTDLQQ SQTVSFAKKT TTTTSTSTTN GWTEGGKISD TLEEKVSVSI 120
                            *         *             *       *        *      120

TIC853    PFIGAGGAKN STTIEANVAH NSSTTTSQQA STEIEWNISQ PVLVPPRKQV VATLVIMGGD 180
Cry51Aa1  PFIGEGGGKN STTIEANFAH NSSTTTFQQA STDIEWNISQ PVLVPPRKQV VATLVIMGGN 183
Cry51Aa2  PFIGEGGGKN STTIEANFAH NSSTTTFQQA STDIEWNISQ PVLVPPSKQV VATLVIMGGN 180
              *  *          *         *      *              *             * 180

TIC853    FTVPMDLITT IDSTQ---HF TGYPILTWIE NPEHNVRGRF LSWFFANWPN LPSEFGSLNS 237
Cry51Aa1  FTIPMDLMTT IDSTE---HY SGYPILTWIS SPDNSYNGPF MSWYFANWPN LPSGFGPLNS 240
Cry51Aa2  FTIPMDLMTT IDSTEHYSHY SGYPILTWIS SPDNSYSGPF MSWYFANWPN LPSGFGPLNS 240
            *    *       **** * *        * * ***** *  *  *          *  *

TIC853    DNTITYKGSV VSRISAGVYA TVRFDQYAIN NLRTIEKTWY ARHGTLHNGK KISINNVTEM 297
Cry51Aa1  DNTVTYTGSV VSQVSAGVYA TVRFDQYDIH NLRTIEKTWY ARHATLHNGK KISINNVTEM 300
Cry51Aa2  DNTVTYTGSV VSQVSAGVYA TVRFDQYDIH NLRTIEKTWY ARHATLHNGK KISINNVTEM 300
             *  *      **              * *               *

TIC853    APTSPIERN  306
Cry51Aa1  APTSPIKTN  309
Cry51Aa2  APTSPIKTN  309
                **   309
```

Supplementary Fig. 4. Alignment of the Cry51Aa1, Cry51Aa2, and TIC853 (ref. 14) amino acid sequences. Amino acid differences are denoted by the asterisk.

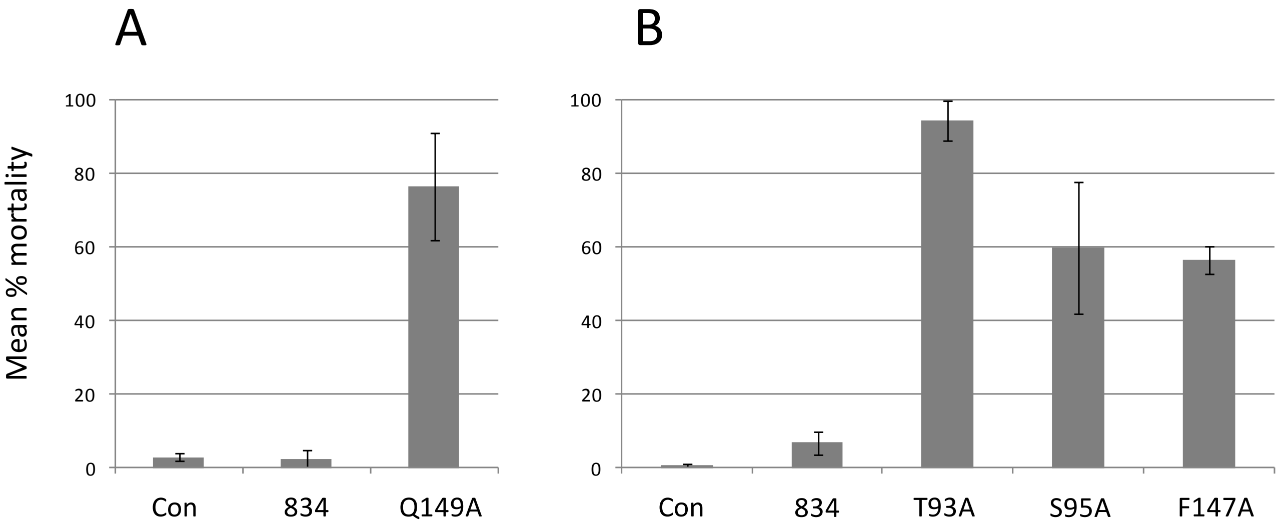

Supplementary Fig. 5. Bioassays demonstrating improved activity of select Cry51Aa2.834 alanine substitution variants towards *L. hesperus.* Proteins were presented in the diet at a concentration of 3.1 ppm. Con = buffer negative control; 834 = parental Cry51Aa2.834 protein. Data shown as mean ± s.e.m. (*n*=24). Panels A and B summarize data from two independent tests.

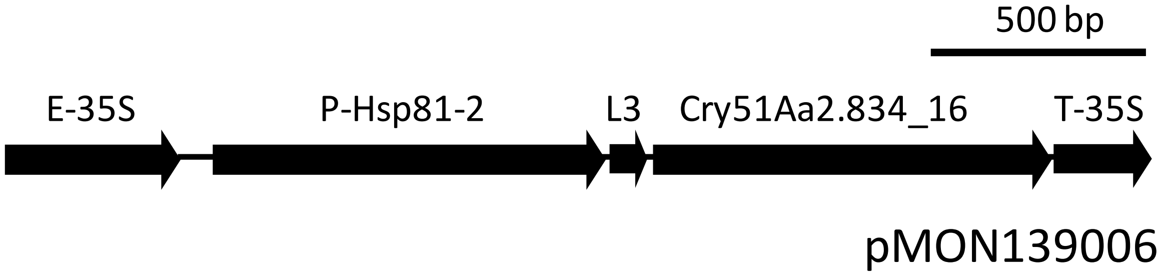

Supplementary Fig. 6. Schematic diagram of the Cry51Aa2.834_16 expression cassette contained on the plant transformation vector pMON139006. E- enhancer, P-Promoter, L- leader and T-terminator.

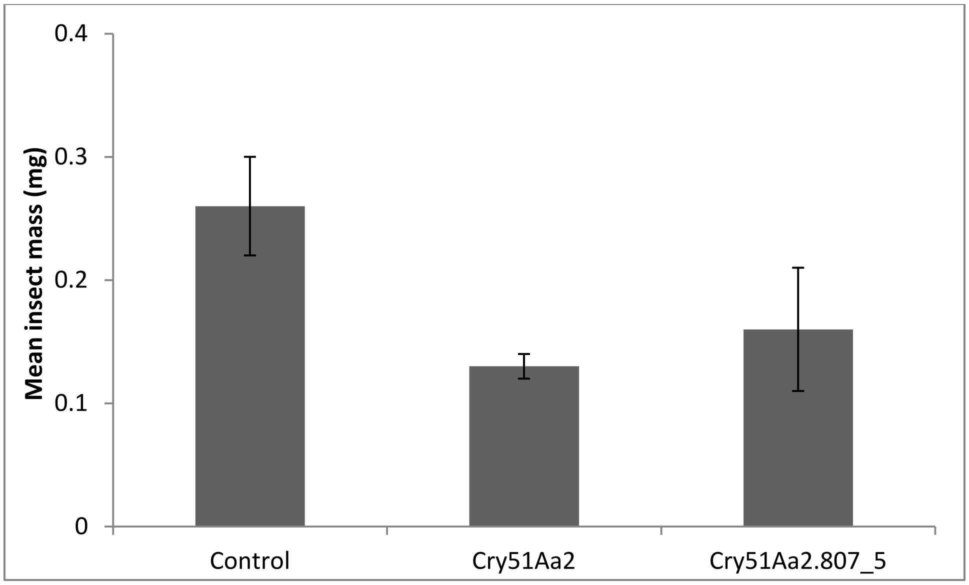

Supplementary Fig. 7. Bioassay demonstrating that the HYS deletion in Cry51Aa2.807_5 protein does not impact activity towards *Lygus* lineolaris. Proteins were presented in the diet at a concentration of 100 ppm and the assay scored for mean insect mass. Control = buffer negative control; Data shown as mean ± s.e.m. (*n*=40).

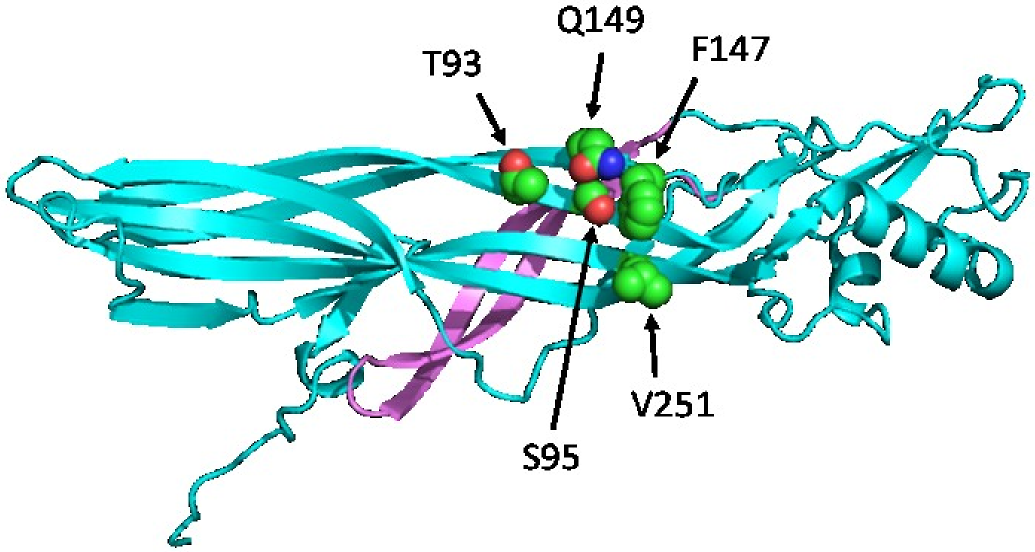

Supplementary Fig. 8. Alanine substitution sites in the wild-type Cry51Aa2 resulting in increased activity towards *Lygus hesperus* are located on three adjacent β strands in close proximity to each other. The putative beta-pore-forming loop is colored magenta.

Supplementary Table 1. Data collection, phasing and refinement statistics for SeMet-Cry51Aa1-L11M: MAD data collection for structure solution and high resolution data for refinement

	Native	Crystal 1		Crystal 2
**Data collection**				
Space group		P43212		P43212
Cell dimensions				
a, *b*, *c* (Å)		54.8, 54.8, 208.9		55.6, 55.6, 209.0
α, β, γ (°)		90°, 90°, 90°		90°, 90°, 90°
	Peak	*Inflection*	*Remote*	
Wavelength	0.97972	0.97996	0.97228	1.00
Resolution (Å)	50-2.75	20-2.80	20-2.8	53.7-2.28
R_{sym} or R_{merge}	0.109	0.110	0.105	0.08
$I / \sigma I$	34.0	32.3	37.3	17.4
Completeness (%)	99.9%	100%	99.9%	95.9%
Redundancy	22.6	22.6	22.6	4.7
**Refinement**				
Resolution (Å)		53.7-2.75		53.7-2.28
No. reflections		9165		15297
R_{work} / R_{free}		0.218/0.304		0.238/0.295
No. atoms		2456		2437
Protein		2390		2390
Ligand/ion		-		-
Water		66		47
B-factors				
Protein		42.1		41.4
Ligand/ion		-		-
Water		31.7		41.3
R.m.s deviations				
Bond lengths (Å)		0.014 Å		0.019 Å
Bond angles (°)		1.60°		1.80°

.

Supplementary Table 2. Cry51Aa2 alanine scan variants showing increased mortality compared to the wild-type Cry51Aa2 protein towards *Lygus hesperus* in artificial diet feeding assays

Amino acid substitution	Strategy	Test concentration (ppm)
S95A, F147A	Combinatorial	12.5
E125A	Alanine scan	25
E125A, F147A	Combinatorial	12.5
E125A, Q149A	Combinatorial	12.5
G128A	Alanine scan	25
I134A	Alanine scan	25
E135S	Alanine scan	25
T145A	Alanine scan	25
T145A, F147A	Double Ala scan	25
F147A	Alanine scan	25
F147A, Q149A	Double Ala scan	25
F147A, S159A	Combinatorial	12.5
F147A, L187A	Combinatorial	12.5
Q149A	Alanine scan	25
Q149A, S151A	Double Ala scan	25
Q149A, T182A	Combinatorial	12.5
D153A	Alanine scan	25
S159A	Alanine scan	25
V175A	Alanine scan	25
T182A	Alanine scan	25
L187A	Alanine scan	25
I123A, A139S	Combinatorial	25
