# Supplementary material for: A transgenic approach for controlling Lygus in cotton
Source: Nat Commun. 2016 Jul 18;7:12213. doi: 10.1038/ncomms12213 (PMC4960306; doi:10.1038/ncomms12213)
Supplement: Supplementary Data 2 — Coding regions for Cry51Aa2 and Cry51Aa2.834 [file ncomms12213-s3.docx]

>Cry51Aa2

ATGGCAATTCTCGACTTAAAGTCTTTAGTCTTGAATGCGATTAATTATTGGGGCCCAAAGAATAACAATGGGATACAAGGCGGTGATTTTGGTTATCCGATTAGTGAGAAACAGATCGATACGAGTATTATTACTTTTACACATCCAAGATTAATTCCCTATGACCTAACAATACCACAAAACTTAGAAACGATCTTTACAACTACACAAGTATTAACAAACAATACGGATTTGCAACAAAGTCAAACAGTATCTTTTGCCAAGAAAACAACCACGACAACGTCAACATCTACGACTAATGGATGGACTGAAGGAGGGAAAATCTCTGATACTCTTGAAGAGAAAGTTAGCGTTAGTATTCCTTTTATTGGAGAAGGAGGTGGTAAGAATTCTACTACAATAGAAGCTAATTTTGCACATAATAGTAGTACAACAACTTTTCAACAGGCTTCCACCGACATTGAATGGAACATCTCACAACCAGTGCTTGTACCACCATCTAAACAGGTAGTAGCTACGCTGGTTATTATGGGTGGTAATTTTACAATACCAATGGATTTGATGACAACTATTGATAGTACGGAGCATTATTCCCATTATTCTGGTTATCCTATTTTAACTTGGATCTCTAGTCCTGATAATAGTTATTCAGGCCCATTCATGAGTTGGTATTTTGCAAATTGGCCGAATCTTCCGAGTGGTTTTGGTCCTTTAAATTCTGATAACACAGTAACGTACACAGGGTCCGTTGTTAGCCAAGTTTCAGCAGGGGTCTATGCGACGGTTCGCTTCGATCAGTATGACATTCATAATCTAAGGACGATTGAGAAAACATGGTACGCAAGACATGCTACTCTTCATAATGGGAAGAAAATTTCTATTAATAACGTTACAGAAATGGCACCTACAAGTCCTATCAAAACAAACTAAGCATGCTGA

>Cry51Aa2.834

ATGGCAATTTTAGATCTTAAAAGTTTAGTACTTAATGCAATAAATTATTGGGGACCTAAAAATAATAATGGAATTCAAGGAGGTGATTTTGGATATCCAATAAGTGAAAAACAAATAGATACTAGTATAATTACTAGTACACATCCTAGATTAATTCCACATGATTTAACAATTCCACAAAATTTAGAAACAATTTTTACAACTACACAAGTATTAACAAATAATACAGATTTACAACAGAGTCAAACAGTAAGTTTTGCAAAAAAGACAACTACAACTACAAGTACAAGTACAACTAATGGATGGACAGAAGGAGGTAAAATTTCTGATACACTTGAAGAGAAAGTAAGCGTAAGCATTCCATTTATTGGAGAAGGAGGTGGAAAAAATAGCACAACTATTGAAGCAAATTTTGCACATAATTCTTCAACAACTACATTTCAACAGGCTTCTACTGATATTGAATGGAATATTTCTCAACCAGTACTAGTACCACCTAGAAAACAAGTTGTGGCTACTCTAGTTATTATGGGTGGGAATTTTACTATACCAATGGATCTAATGACTACGATAGATTCTACTGAACATTATTCTGGTTATCCAATATTGACGTGGATATCTTCACCTGATAATTCATATAATGGTCCTTTTATGTCATGGTATTTCGCTAATTGGCCTAATTTGCCGTCAGGGTTCGGGCCGTTGAATTCAGACAACACGGTTACGTATACGGGGTCCGTTGTGTCCCAAGTTTCCGCTGGCGTGTATGCTACGGTCAGGTTCGACCAGTACGACATCCATAACCTGCGTACCATCGAGAAAACCTGGTACGCGCGACATGCGACCCTCCACAACGGCAAAAAGATCTCGATCAACAACGTCACCGAGATGGCCCCGACCTCGCCCATCAAGACCAACTAA
